# Supplementary material for: Development of a computer-aided design software for the quantitative evaluation of aesthetic damage
Source: PLoS One. 2019 Dec 18;14(12):e0226322. doi: 10.1371/journal.pone.0226322 (PMC6919621; doi:10.1371/journal.pone.0226322)
Supplement: S1 Code site — 1. (ZIP) [file pone.0226322.s001.zip › [EESC_jr][Projeto]Relatorio_07_21/vendors/select2/docs/_includes/examples/programmatic-control.html]

# Programmatic control

## DOM events

Select2 will trigger some events on the original select element,
allowing you to integrate it with other components. You can find more
information on events
on the options page.

`change` is fired whenever an option is selected or removed.

`select2:open` is fired whenever the dropdown is opened.
`select2:opening` is fired before this and can be prevented.

`select2:close` is fired whenever the dropdown is closed.
`select2:closing` is fired before this and can be prevented.

`select2:select` is fired whenever a result is selected.
`select2:selecting` is fired before this and can be prevented.

`select2:unselect` is fired whenever a result is unselected.
`select2:unselecting` is fired before this and can be prevented.

## Programmatic access

Select2 supports methods that allow programmatic control of the
component.

Set "California"

Open

Close

Init

Destroy

Set to California and Alabama

Clear
